# Conversion of Organic Dyes into Pigments: Extraction of Flavonoids from Blackberries (*Rubus ulmifolius*) and Stabilization

**DOI:** 10.3390/molecules26206278

**Published:** 2021-10-17

**Authors:** Rossella G. Candela, Giuseppe Lazzara, Sonia Piacente, Maurizio Bruno, Giuseppe Cavallaro, Natale Badalamenti

**Affiliations:** 1 Department of Biological, Chemical and Pharmaceutical Sciences and Technologies (STEBICEF), University of Palermo, Viale delle Scienze, 90128 Palermo, Italy; r.gaglianocandela88@gmail.com (R.G.C.); maurizio.bruno@unipa.it (M.B.); 2 Physics and Chemistry Department (DiFC), University of Palermo, Viale delle Scienze, 90128 Palermo, Italy; giuseppe.lazzara@unipa.it; 3 Department of Pharmacy, University of Salerno, 84084 Fisciano (SA), Italy; piacente@unisa.it

**Keywords:** blackberries, *Rubus ulmifolius*, anthocyanin, pigments, LC-ESI/LTQOrbitrap/MS, thermogravimetry, colorimetric analysis

## Abstract

The blackberry’s color is composed mainly of natural dyes called anthocyanins. Their color is red–purple, and they can be used as a natural colorant. Anthocyanins are flavonoids, which are products of plants, and their colors range from orange and red to various shades of blue, purple and green, according to pH. In this study, the chemical composition of an extract obtained from blackberries was defined by LC-ESI/LTQOrbitrap/MS in positive and negative ionization mode. Furthermore, we investigated the adsorption process of blackberry extract using several inorganic fillers, such as metakaolin, silica, Lipari pumice, white pozzolan and alumina. The pigments exhibit different colors as a function of their interactions with the fillers. The analysis of the absorption data allowed the estimation of the maximum adsorbing capacity of each individual filler tested. Through thermogravimetric measurements (TGA), the thermal stability and the real adsorption of the organic extract were determined.

## 1. Introduction

The colors of flowers and fruits derive essentially from the absorption of visible light, but also from various secondary substances synthesized by higher plants. The molecules responsible for the colors of fruit are essentially flavonoids, distributed inside the vacuoles [1]. They represent a large class of phenolic compounds including flavones, chalcones, aurones and anthocyanidins. Anthocyanins are mainly responsible for the pink, orange, red, purple and blue flowers, fruit and vegetables [2]. Some chalcones, aurones and flavonols contribute, for example, to the yellow color; most of the remaining flavones, flavonols and their glycosylates are, however, colorless to humans, but act as co-pigments and can be visible to some insects [3].

**Figure 1:**
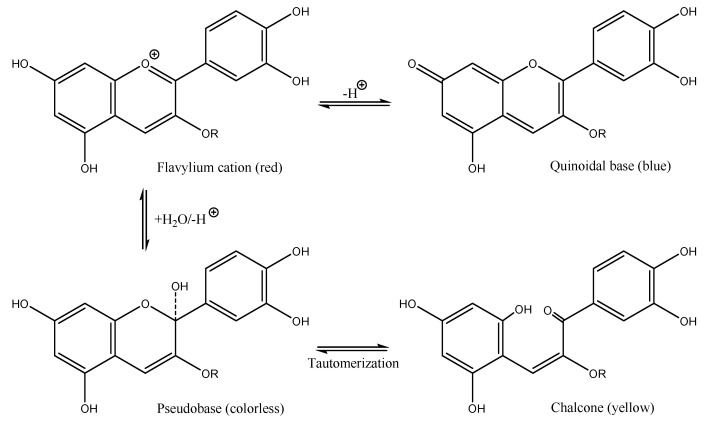
The chemical structures of some anthocyanins based on pH.

However, the major contribution to the coloring is given by anthocyanins. These are a class of water-soluble dyes called E163 in the European Coding of Food Additives [4]. The choice of an extraction method is of great importance in the analysis of anthocyanins, and largely depends on the purpose of the use, the nature of the anthocyanins and the source material. Recent studies have shown that anthocyanins can be used as natural food colorants and dyes for fabrics [5,6,7]. However, since anthocyanins are very unstable molecules, their applications are limited in other fields [8]. The scientific literature offers a considerable number of articles concentrated on the study of anthocyanins obtained from different plant species. The extraction methods of anthocyanins are mainly based on the use of a single solvent [9], or different solvents [10], but in the last decade, green methods, without the aid of solvents and with shorter extraction times, such as extraction assisted by enzymes [11], or by ultrasound [12] or a microwave [13], have largely taken hold. In a very interesting article [14], the different methods of characterization of anthocyanins (high-performance liquid chromatography with a diode-array detector (HPLC-DAD), with mass spectrometry (HPLC-DAD-MS) or with tandem mass spectrometry (HPLC-DAD-MS/MS)) were also considered, and the beneficial effects on health in relation to the structure–activity mechanism were considered. In this study, we studied how to stabilize anthocyanins by incorporating them into an inorganic matrix with a certain pH and how to use them as pigments. pH has a significant impact on anthocyanin molecules. In fact, this determines the color and chemical form of an anthocyanin. At a pH below 2, anthocyanins exist in some cationic form (purple and red flavylium cation). By increasing the pH up to 4, the quinoidal blue species are predominant, and between pH 5 and 6, the color turns yellow [15,16,17] (Figure 1). In addition to pH, the color stability of anthocyanins is significantly affected by chemical structure, solvents, temperature, concentration, oxygen and light [18,19]. Higher anthocyanin concentrations result in more stability and intensity of color [20,21]. It is of fundamental importance to choose the right acid or basic matrix according to the color you wish to obtain. In this study, we had to obtain hot colors, despite the original color of the blue raspberry, by encapsulating them in several inorganic compounds.

The problem regarding stability is very serious and prevents the widespread use of any anthocyanin as a colorant other than for food dyeing. The incorporation of organic dyes into inorganic host materials has been widely investigated [22,23,24,25], and this has been done by ancient people—take the blue Egyptian and Mayan blue pigments, where indigo is incorporated in the pores of palygorskite clay [26,27]. There have been many papers concerning enhancement of the stability of organic dyes by complexation with inorganic host materials such as clays [28,29,30,31,32,33,34] and zeolites [35,36,37,38,39]. The stabilization of anthocyanins, as a function of pH, has involved various material applications: their use as dyes for silk, for renewable hair, for cosmetics or as colors for commercial products in the food sector [17,20,40,41]. Furthermore, stabilized pigments obtained by transforming an organic dye (based on anthocyanins) into an inorganic one, can be used in the field of restoration in the form of pictorial retouching [42]. As reported in our previous article [43], the adsorption capacity of inorganic fillers for functional organic molecules can be correlated to the thermal behavior of the corresponding composite materials. In this regard, it should be noted that thermogravimetric analysis represents a powerful technique to investigate the thermal characteristics of inorganic–organic hybrids, which include polymeric nanocomposites [44,45,46,47], nanocarriers [48,49,50] and catalytic supports [51,52]. The interpretation of the thermogravimetric data can be helpful for exploring the mechanisms of interaction between inorganic fillers and organic molecules, allowing one to control the properties and the consequent potential applications of the hybrid materials [53,54].

Consequently, in the context of our ongoing research on Sicilian plants [55,56,57,58,59] and possible applications [60,61,62,63,64], we sought to confirm the absorption of flavonoids from Sicilian *Rubus* by different inorganic matrices, such as metakaolin, silica, Lipari pumice, white pozzolana and alumina. Colorimetric analysis, adsorption tests and thermogravimetric analysis were performed to evaluate possible host–guest interactions, adsorbent capacity and thermal stability of the *Rubus ulmifolius* extract on the different inorganic fillers.

## 2. Results

### 2.1. LC-ESI/LTQOrbitrap/MS/MS^n^ Analysis

Samples were analyzed by liquid chromatography-electrospray ionization/linear triple quadrupole orbitrap/mass spectrometry (LC-ESI/LTQOrbitrap/MS/MS^n^) in positive and negative ionization modes, and the identities of the peaks were attributed according to their accurate masses, characteristic fragmentation patterns and retention times; literature on *R. ulmifolius*; and whenever possible, by comparison with standards (Table 1).

A detailed screening of the chemical profiles highlighted the presence of anthocyanins, flavonol glycosides, quercetin, taxifolin (dihydroquercetin) glucoside and taxifolin pentoside, along with proanthocyanidins and chlorogenic acid.

**Figure 2:**
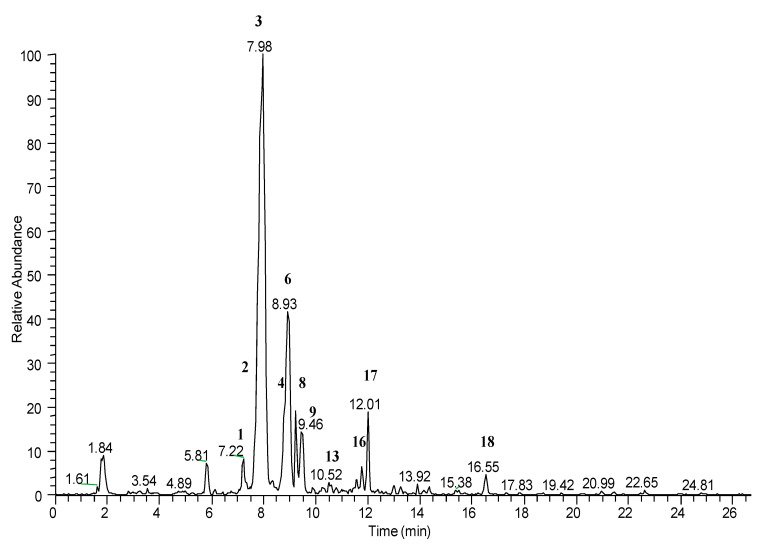
LC-ESI/LTQOrbitrap/MS profile in positive ion mode.

The analysis of the LC-ESI/HR/MS profile obtained in positive ionization mode was crucial to highlight the occurrence of anthocyanins (Figure 2). In particular, it revealed the presence of six anthocyanins (**2**, **3**, **4**, **6**, **9** and **13**). The fragmentation pattern of compound **2** showed the subsequent losses of two hexose units (162 amu) from the molecular ion at *m/z* 611.1591, generating a peak at *m/z* 287 corresponding to the aglycone, identified as cyanidin. This peak was observed in the fragmentation patterns of compounds **3**, **6** and **9** too, identifying them as cyanidin glycosides.

In particular, the molecular ion of **3** at *m/z* 449.1059 showed the loss of 162 amu corresponding to a hexose unit; the molecular ion of **6** at *m/z* 419.0962 showed the loss of 132 amu corresponding to a pentose unit; and the molecular ion of **9** at *m/z* 593.1481 exhibited a loss of 306 amu attributed to a dioxalylhexoside unit. Compounds **2** and **9** were putatively identified as cyanidin dihexoside and cyanidin dioxalylglucoside already reported in *Rubus* fruit by Primo da Silva et al. [65].

The molecular ions of **4** at *m/z* 433.1117 and **13** at *m/z* 465.1016, after the loss of a hexose unit (162 amu), gave fragment ions at *m/z* 271 and 303 corresponding, respectively, to pelargonidin and delphinidin.

Compounds **3**, **6**, **4** and **13** were identified and confirmed by commercial standards as cyanidin 3-*O*-glucoside, cyanidin 3-*O*-xyloside, pelargonidin 3-*O*-glucoside and delphinidin 3-*O*-glucoside, respectively.

The LC-ESI/HR/MS profile obtained in positive ionization mode showed the occurrence of chlorogenic acid (**1**) confirmed with a commercial standard.

**Figure 3:**
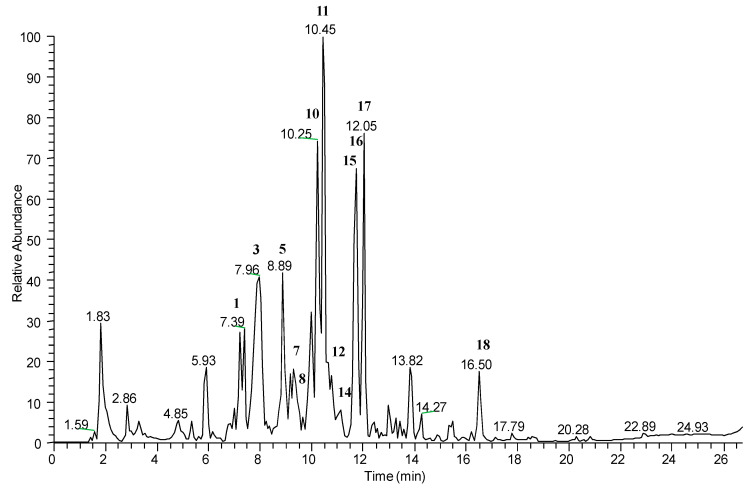
LC-ESI/LTQOrbitrap/MS profile in negative ion mode.

Peaks corresponding to flavonol glycosides, dihydroflavonol glycosides, quercetin and proanthocyanidins could be better observed in the LC-ESI/HR/MS profile obtained in negative ionization mode (Figure 3). In particular, the molecular ion at *m/z* 301.0333 of compound **18** could be easily attributed to quercetin; and the molecular ions at *m/z* 447.0553 (**12**), 463.0868 (**14**) and 477.0641 (**16**) generating the ion at *m/z* 301 by loss of a deoxyhexose unit (146 amu), a hexose unit (162 amu) and a glucuronic acid moiety (176 amu) respectively, could be attributed to quercetin glycosides. Furthermore, the molecular ion at *m/z* 533.0903 showed a fragmentation pattern attributable to kaempferol malonyl glucoside (**7**); and peaks at *m/z* 447.0923 and 593.1490 could be attributed to kaempferol 3-*O*-glucoside (**15**) and kaempferol 3-*O*-rutinoside (**17**), respectively.

Taxifolin pentoside (**8**) at *m/z* 465.1025 and taxifolin glucoside (**5**) at *m/z* 435.0909 could be putatively identified.

Compound **10** exhibited in HRMS a quasi-molecular ion [M − H]^−^ at *m/z* 863.1806, and in the MS/MS spectrum two product ions [M – H − 288]^−^ at *m/z* 575 and [M – H − 290]^−^ at *m/z* 573, generated by the quinone methide fissions of the linkages of (epi)catechin terminal units, linked to a central (epi)catechin moiety by a B-type and an A-type linkage, respectively, allowing us to deduce the occurrence of a proanthocyanidin, namely (epi)catechin-B-(epi)catechin-A-(epi)catechin.

Compound **11** showed a precursor ion [M – H]^−^ at *m/z* 575.1180. In the MS/MS spectrum, it exhibited a base peak [M – H − 126]^−^ at *m/z* 449 generated by the heterocyclic A-ring fission, together with a fragment ion [M – H − 286]^−^ at *m/z* 289 due to the quinone methide fission of the A-type linkage, supporting the putative identification of compound **11** as A-type (epi)catechin dimer [66]. Some mass spectra are enclosed in Supplementary Materials.

### 2.2. Adsorption Isotherms

The adsorption capacities of several nanomaterials (Table 2) toward flavonoids were investigated.

**Figure 4:**
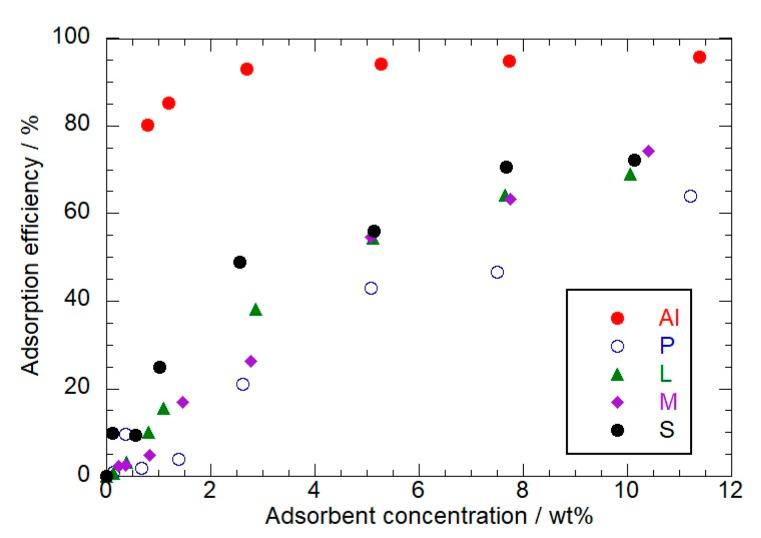
Percentages of flavonoids adsorbed as functions of the filler concentration.

The adsorption experiments were conducted with variable concentrations of the adsorbent nanomaterials, while the temperature was kept constant at 25 °C overnight under magnetic stirring. In particular, the concentrations of the inorganic fillers were systematically changed up to ca. 10 wt%. The obtained adsorption isotherms are presented in Figure 4.

Compared with the other nanofillers, we detected that Al possesses the strongest adsorption efficiency and the highest affinity towards the organic dyes. Table 3 reports the proportions of flavonoids adsorbed (estimated) at the filler concentration of ca. 10 wt%. The corresponding hybrid nanomaterials were investigated by colorimetry and thermogravimetric analysis.

### 2.3. Colorimetric Parameters of the Hybrid Nanomaterials

The colorimetric parameters of the hybrids are collected in Table 4.

We observed that Al-F possesses the lowest L* value (25.59) compared with the other loaded fillers, which presented L* equal to ca. 65. On this basis, we can state that the lightness of neutral alumina filled with flavonoids is significantly inferior with respect to the other composite nanomaterials.

**Figure 5:**
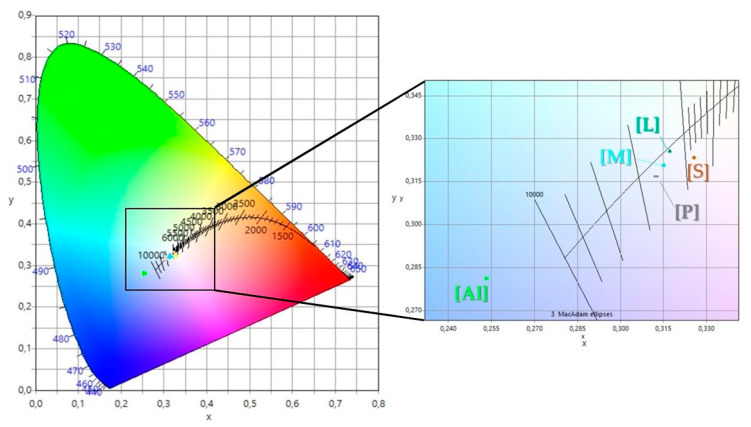
Colorimetric parameters displayed on a CIE diagram, including the sRGB region for the inorganic fillers loaded with flavonoids.

The largest ΔE value was estimated for Al. L showed the slightest color difference compared with the white surface. Figure 5 presents the colorimetric parameters of the hybrid in the CIE diagram, which provides information on the chromaticity of the nanomaterials. The colorimetric parameters are consistent with the strongest efficiency capacity of neutral alumina towards the organic dye (Table 3).

### 2.4. Thermal Properties of the Hybrid Nanomaterials

**Figure 6:**
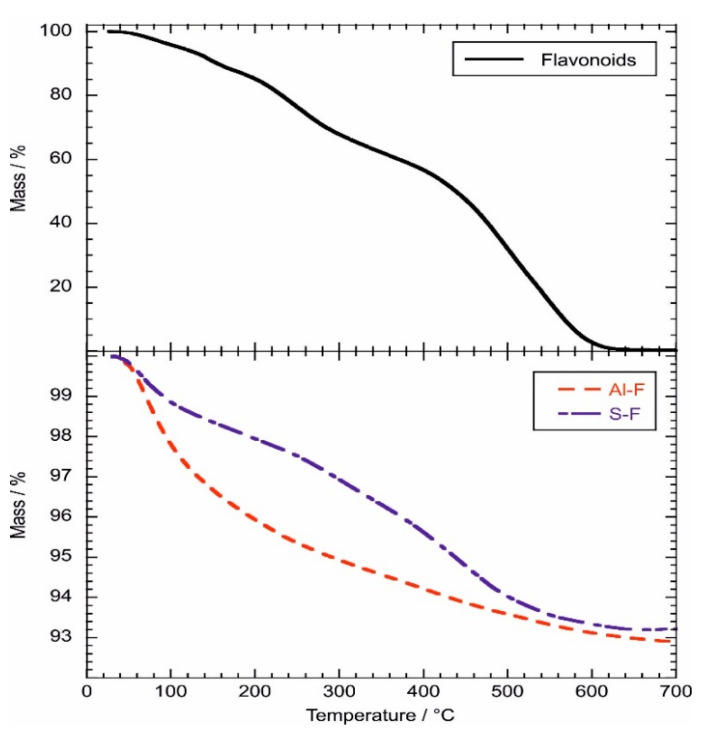
Thermogravimetric curves of pure flavonoids, and Al-F and S-F hybrids.

The thermal characteristics of the hybrid nanomaterials were studied by thermogravimetry, which is a powerful technique with which to investigate composites based on inorganic nanoparticles filled with functional organic molecules [67,68]. As examples, Figure 6 shows the thermogravimetric (TG) curves of Al-F, L-F and pure extract.

Based on the quantitative analysis of the TG curves, we calculated the water contents of the materials from the mass loss occurring between 25 and 150 °C (ML_150_). The obtained data (Table 5) evidenced that Al-F possesses the strongest hydrophilic behavior. On the other hand, L-F is the most hydrophobic hybrid material. As shown in Figure 6, flavonoids were almost completely decomposed within the investigated temperature range. Namely, we calculated a negligible residual mass at 700 °C (MR_700_).

Table 4 evidences that the largest MR_700_ was estimated for M-F. Compared with the other hybrid materials, Al-F resulted in a smaller MR_700_ value. This result is consistent with the largest ΔE value (Table 4) and the strongest adsorption efficiency (Table 3), which were determined by the colorimetric analysis and spectrophotometry, respectively. The thermogravimetric curve of pure flavonoids shows two mass losses in the temperature intervals 200–300 °C and 400–600 °C, indicating that the thermal degradation of the molecules occurs in two separate steps. The presence of a cathecol unit in the extract of *Rubus ulmifolius*, along with natural anthocyanins, allow complexation by trivalent aluminum. This metal, an inorganic element, is not available in a soluble cationic form, but it can be used as a model cation, due to its strong affinity for the cathecol unit. The complexation, already reported in the literature [69], takes place between the metal and the ionized quinoidal base, allowing stabilization of the bluish color. In the absence of the metal, the color is short-lived. The bathochromic shift, caused by the absorption of anthocyanins, was not evident, however, for all the other fillers used since the latter are completely devoid of acidic sites such as Al^3+^ or Mg^2+^ [41].

## 3. Materials and Methods

### 3.1. Material

Fresh blackberries (*Rubus ulmifolius* Schott) were harvested from an agricultural property in Alimena, Sicily, Italy, 80 km south-east of Palermo (37°40′50″ N 14°05′01″ E; 645 m m.s.l.), in September 2018. The fruits were harvested in a single collection at fully mature stages from ten randomly selected plants. The fruits were packed in polyethylene bags with the atmosphere modified with gaseous nitrogen, hermetically sealed and immediately frozen at −24 °C. The botanical identification of *Rubus ulmifolius* Schott was confirmed by Prof. Emanuele Schimmenti, and a sample was deposited in the Department STEBICEF, University of Palermo, Italy, under identification number MB 548/2018.

Neutral alumina (white powder; particle size: range 70–290 mesh; pH range: 7.0–7.5; surface area: regular 155 m^2^/g; % slurry: 5%), metakaolin (grayish powder; particle size: 400-500 mesh; pH range: 5.0–6.0; surface area: 39 m^2^/g; % slurry: < 1%) and silica (white powder; nanoparticles; mesoporous; particle size: 190–250 nm; average pore diameter: 3.5–4.5 nm) were from Sigma-Aldrich (St. Louis, MO 68178, USA); VCAS™ White Pozzolans (white powder; particle size: 325 mesh; pH range: 9.5–10.0; surface area: 0.60 m^2^/g; % slurry: 1%) were from Vitro Minerals, Inc (95 Pinnacle Drive, Jackson, TN 38301, USA); Lipari pumice (white porous powder; particle size: 220–350 mesh; pH range: 7.3–8.2; surface area: 210 m^2^/g; slurry: 2–3%) was from NOVA SPA (Via Fossanuova 55, 55016 Porcari (LU), Italy). All the products were used without any further purification. Standards for LC-ESI/LTQOrbitrap/MS analysis were from Sigma-Aldrich (St. Louis, MO 68178, USA).

### 3.2. The Extraction Procedure for the Flavonoids

The frozen blackberries were lyophilized, and 50 g of dry material was blended with 400 mL of water and acetone (30:70) in a Waring^®^ Blender (Thermo Fisher Scientific, Waltham, Massachusetts, USA). The volume of the mixture was reduced at low pressures and finally transferred to a separating funnel. Then, 400 mL of chloroform was added. As a result, two phases were created, a water/blue raspberry mixture (which contained anthocyanins, phenolic compounds, sugars, organic acids and other water-soluble compounds) and an immiscible chloroform/acetone layer (lipids, carotenoids, chlorophyll pigments and others non-polar compounds) [70]. The colored aqueous layer was filtered again by an Büchner flask to remove insoluble parts. The filtered aqueous layer was subsequently purified from the presence of sugars by liquid chromatography on a column, using C_18_-Reversed phase silica gel (Fluka^®^ Analytical) (5.125 g) as the stationary phase. To cause the main elution of the sugars, two solutions were prepared: acidic water (0.01 M, pH = 2) and acidic methanol (0.01 M, 8 µL of 37 % HCl in 100 mL of methanol). Once the glucosidic impurities were eliminated by repeated elution with the aqueous acid solution (3 × 14 mL), the extract containing the flavonoids was obtained by eluting it with the acidic methanolic mixture. From 50 g of blackberries was collected 200 mL of colored extract in acidic methanol without contaminants, containing 1.15 g of organic metabolites (yield 2.30%).

### 3.3. LC-ESI/LTQOrbitrap/MS/MSn Analysis

Qualitative LC-MS analysis was performed by using an Accela HPLC system (Thermo Scientific, Germany), equipped with a Phenomenex Luna C18 column (150 mm × 2.1 mm i.d., 5 μm) and working at a flow rate of 0.2 mL/min, coupled to an LTQ-Orbitrap XL mass spectrometer (Thermo Fisher Scientific, San Jose, CA, USA). The Orbitrap mass analyzer was calibrated according to the manufacturer’s directions using a mixture of caffeine, methionine-arginine phenylalanine-alanine-acetate (MRFA), sodium dodecyl sulfate, sodium taurocholate and Ultramark 1621. Data were collected and analyzed using the software provided by the manufacturer. Samples were prepared as 1 mg/mL (methanol/water), and 5 μL of each was injected and analyzed in negative and positive ionization modes following a protocol previously reported, with slight modifications [71]. The following solvent system was used: H_2_O + 0.1% formic acid (A) and CH_3_CN 0.1% formic acid (B). The gradient program was: 0–30 min, from 5 to 95% (B); then 5 min at 95% (B); and back to 5% (B) for 5 min; flow rate: 0.2 mL/min. ESI source parameters in negative ion mode: capillary voltage −48 V; tube lens voltage −176.47; capillary temperature 280 °C; sheath and auxiliary gas flow (N_2_), 15 and 5; sweep gas 0; spray voltage 5. In positive ion mode: capillary voltage 49 V; tube lens voltage 120; capillary temperature 280 °C; sheath and auxiliary gas flow (N_2_), 30 and 5; sweep gas 0; spray voltage 5. The full range acquisition covering *m/z* 120–1600 was used for both the polarities. A fragmentation study was performed by using data dependent scan mode, while selecting precursor ions corresponding to most intense peaks in LC-MS spectra. Xcalibur software version 2.1 was used for instrument control, data acquisition and data analysis.

### 3.4. Colorimetric Analysis

The color parameters of the materials were measured using a colorimeter (NH300 Colorimeter, 3NH Shanghai Co., Ltd.). Black and white plates were used to calibrate the instrument. CQCS3 software was used for data analysis and results. The total color differences (*ΔE*), the parameters a* (red–green), L* (lightness) and b* (yellow–blue), were measured for each material obtained and compared with a white surface used as a reference parameter, as the norm dictates.

### 3.5. Spectrophotometry

Spectrophotometric experiments were conducted to investigate the adsorption, which provided the adsorption capacities of the fillers (neutral aluminum oxide, white pozzolana, metakaolin, Lipari pumice and neutral slice) toward flavonoid fraction. The experiments were carried out using an Analytik Jena Specord S600 BU in isothermal conditions. (The temperature was set to 25.0 ± 0.1 °C.) The methanol solution of the extract exhibited the maximum of the absorption band at 538.5 nm. Referring to various scientific works [32,33,34], blackberries’ flavonoids/filler dispersions were prepared and equilibrated for 7 days. The concentration of extract was kept constant (0.1 g mL^−1^), while the contents of fillers were systematically changed. Then, the absorbance of the supernatant was measured. The percentage of absorption was estimated through the following Equation:

A% = 10^2^ × (C_Qi_ − C_Qf_) / (C_Qi_) (1)

where C_Qi_ is the initial concentration of the extract solution, and C_Qf_ corresponds to the concentration of extract in the supernatant after the equilibration with the fillers.

### 3.6. Thermogravimetry

The measurements were performed using a Q5000 IR apparatus (TA Instruments) under a nitrogen flow of 25 cm^3^ min^−1^ for the sample and 10 cm^3^ min^−1^ for the balance. The samples (ca. 6 mg) were heated from 25 to 700 °C. The heating rate was set at 20 °C min^−1^. TG analyses were performed on the fillers loaded with flavonoid fractions and on the corresponding pristine components. The temperature calibration of the apparatus was conducted based on the Curie temperatures of standards (nickel, cobalt and their alloys) [72].

## 4. Conclusions

Natural organic molecules from plant matrices, such as *Rubus ulmifolius*, have been used as organic dyes and incorporated into different inorganic host materials (metakaolin, silica, Lipari pumice, white pozzolan and alumina neutral). Colorimetric analysis, the adsorption tests and the thermogravimetric analysis were performed to evaluate the possible organic–inorganic interactions, adsorbent capacity and thermal stability of flavonoids with the several inorganic fillers. The absorption curves showed that, compared to other nanofillers, neutral alumina has the strongest affinity and the greatest adsorption efficiency towards the organic dye. The chelating capacity of alumina against natural pigments, due to the presence of the Al^3+^ acid site, was also confirmed by the determination of the different colorimetric parameters (L, a, b and E) clearly different from all the other inorganic–organic hybrids used. In fact, net bathochromic effects were not visible with the other fillers studied. Finally, quantitative thermogravimetric analysis showed that the greatest organic loading was obtained for the Al-based hybrid nanomaterial, confirming the strongest adsorption efficiency and the largest ΔE value.

## Figures and Tables

**Table 1:** Secondary metabolites identified in the extract of blackberries fruits by LC-ESI/LTQOrbitrap/MS/MS^n^ analysis, operating in positive and negative ion modes.

N°	Rt ^a^	[M + H]^+ b^ [M]^+ c^	[M − H]^− d^	Molecular Formula	MS/MS	Identity
1	7.22	355.1012		C_16_ H_18_ O_9_	163.04	chlorogenic acid *
2	7.40	611.1591		C_27_ H_31_ O_16_	449.11/287.05	cyanidin dihexoside
3	7.98	449.1059		C_21_ H_21_ O_11_	287.05	cyanidin 3-*O*-glucoside *
4	8.56	433.1117		C_21_ H_21_ O_10_	271.06	pelargonidin 3-*O*-glucoside *
5	8.89		465.1025	C_21_ H_22_ O_12_	303.05	taxifolin glucoside
6	8.93	419.0962		C_20_ H_19_ O_10_	287.05	cyanidin 3-*O*-xyloside *
7	9.13		533.0903	C_24_ H_22_ O_14_	447.11/285.06	kaempferol malonyl glucoside
8	9.37		435.0909	C_20_ H_20_ O_11_	303.06	taxifolin pentoside
9	9.46	593.1481		C_27_ H_28_ O_15_	287.05	cyanidin dioxalylglucoside
10	10.25		863.1806	C_45_ H_36_ O_18_	575.09/573.87	(epi)catechin-B-(epi)catechin-A-(epi)catechin
11	10.45		575.1180	C_30_ H_24_ O_12_	449.07/289.21	(epi)catechin-A-(epi)catechin
12	10.48		447.0553	C_20_ H_16_ O_12_	301.03	quercetin 3-*O*- rhamnoside
13	10.52	465.1016		C_21_ H_21_ O_12_	303.05	delphinidin 3-*O*-glucoside *
14	11.61		463.0868	C_21_ H_20_ O_12_	301.03	quercetin 3-*O*-glucoside *
15	11.68		447.0923	C_21_ H_20_ O_11_	285.04	kaempferol 3-*O*-glucoside *
16	11.77		477.0641	C_21_ H_18_ O_13_	301.05	quercetin glucuronide
17	12.05		593.1490	C_27_H_30_O_15_	285.05	kaempferol 3-*O*-rutinoside *
18	16.50		301.0333	C_15_ H_10_ O_7_	-	quercetin

^a^ Retention times. ^b^ Molecular ion in positive ion mode for chlorogenic acid (**1**). ^c^ Molecular ion in positive ion mode for anthocyanins (**2**, **3**, **4, 6**, **9**, **13**). ^d^ Molecular ions in negative ion mode. * Compounds identified with standards.

**Table 2:** Materials used for the adsorption tests of flavonoids.

Acronym	Nanofiller
Al	Neutral alumina
P	White pozzolan
L	Lipari pumice
M	Metakaolin
S	Silica

**Table 3:** Adsorption efficiency for nanofiller concentrations of ca. 10 *wt*%.

Nanofiller	Adsorption Efficiency / wt%
Al	95.6
P	63.8
L	68.9
M	74.2
S	72.1

**Table 4:** Colorimetric parameters of the hybrid nanomaterials.

Hybrid Nanomaterial	L *	a *	b *	ΔE *
Al-F	25.59	−3.16	−10.74	11.32
P-F	65.94	4.47	−3.48	6.07
L-F	66.91	2.87	−0.35	3.25
M-F	67.83	4.13	−2.05	5.02
S-F	63.60	6.71	0.16	7.05

* Control: white paper, L = 97.17, a = −0.34, b = 0.22.

**Table 5:** Thermogravimetric parameters for flavonoids and hybrid nanomaterials.

Material	ML_150_ / wt%	MR_700_ / wt%
Flavonoids	9.34	0.3
Al-F	3.32	92.9
P-F	0.78	95.3
L-F	0.11	95.4
M-F	0.58	97.4
S-F	1.66	93.2

## Data Availability

Data is contained within the article.

## Supplementary Materials

The following are available online. Figure S1: MS/MS spectrum of compound **3** (cyanidin 3-*O*-glucoside), Figure S2: MS/MS spectra of compound **4** (pelargonidin 3-*O*-glucoside), Figure S3: MS/MS spectra of compound **6** (cyanidin xyloside), Figure S4: MS/MS spectra of compound **13** (delphinidin 3-*O*-glucoside), Figure S5: MS/MS spectra of compound **14** (quercetin 3-*O*-glucoside), Figure S6: MS/MS spectra of compound **15** (kaempferol 3-*O*-glucoside).

## Sample Availability

Samples of the compounds are available from the authors.
