# Supplementary material for: Conversion of Organic Dyes into Pigments: Extraction of Flavonoids from Blackberries (Rubus ulmifolius) and Stabilization
Source: Molecules. 2021 Oct 17;26(20):6278. doi: 10.3390/molecules26206278 (PMC8538118; doi:10.3390/molecules26206278)
Supplement: Supplementary file 1 [file molecules-26-06278-s001.zip › molecules-1405488-supplementary.pdf]

Supporting Material

Conversion of Organic Dyes into Pigments: Flavonoids Extraction from Blackberries (*Rubus ulmifolius*) and Stabilization

Rossella Gagliano Candela [1], Giuseppe Lazzara [2], Sonia Piacente [3], Maurizio Bruno [1], Giuseppe Cavallaro [2,*] and Natale Badalamenti [1,*]

[1] Department of Biological, Chemical and Pharmaceutical Sciences and Technologies (STEBICEF), University of Palermo, Viale delle Scienze, Parco d'Orleans II, 90128, Palermo, Italy;
[2] Physics and Chemistry Department (DiFC), University of Palermo, Viale delle Scienze, Palermo d'Orleans II, 90128, Palermo, Italy;
[3] Department of Pharmacy, University of Salerno, Fisciano (SA), 84084, Italy
* Correspondence: giuseppe.cavallaro@unipa.it; natale.badalmenti@unipa.i

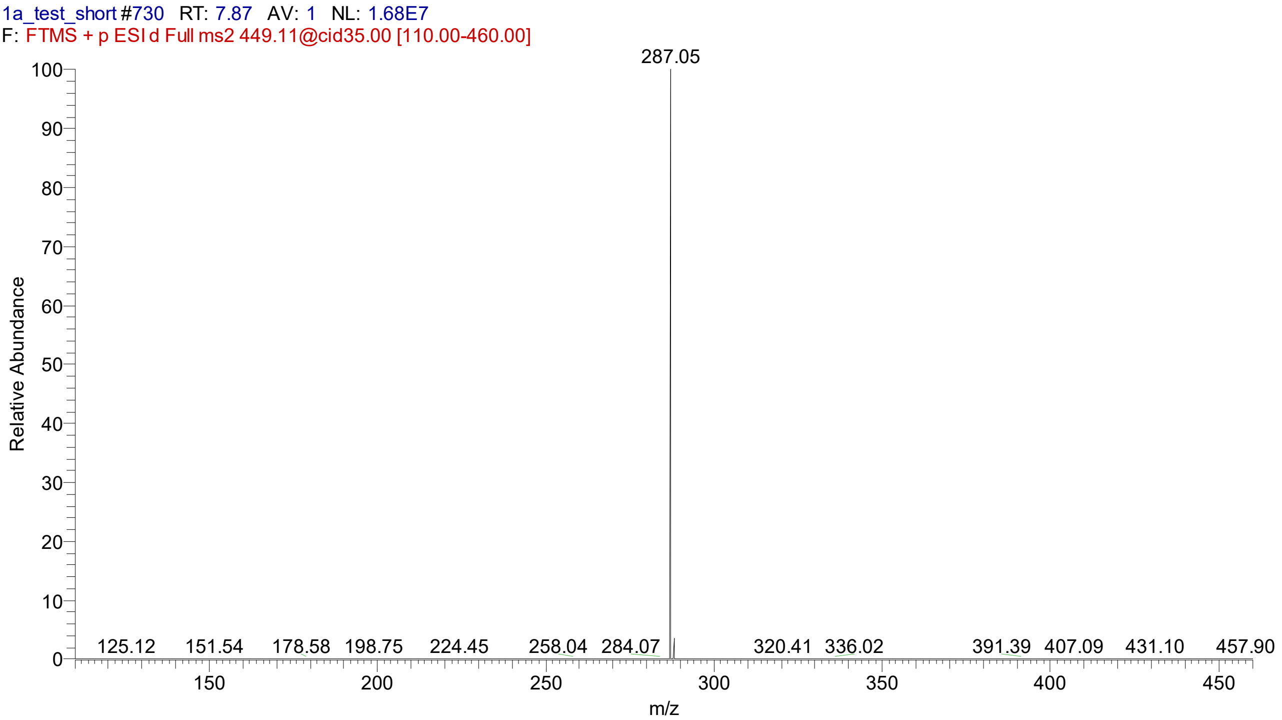

Figure S1. MS/MS spectrum of compound **3** (cyanidin 3-*O*-glucoside)

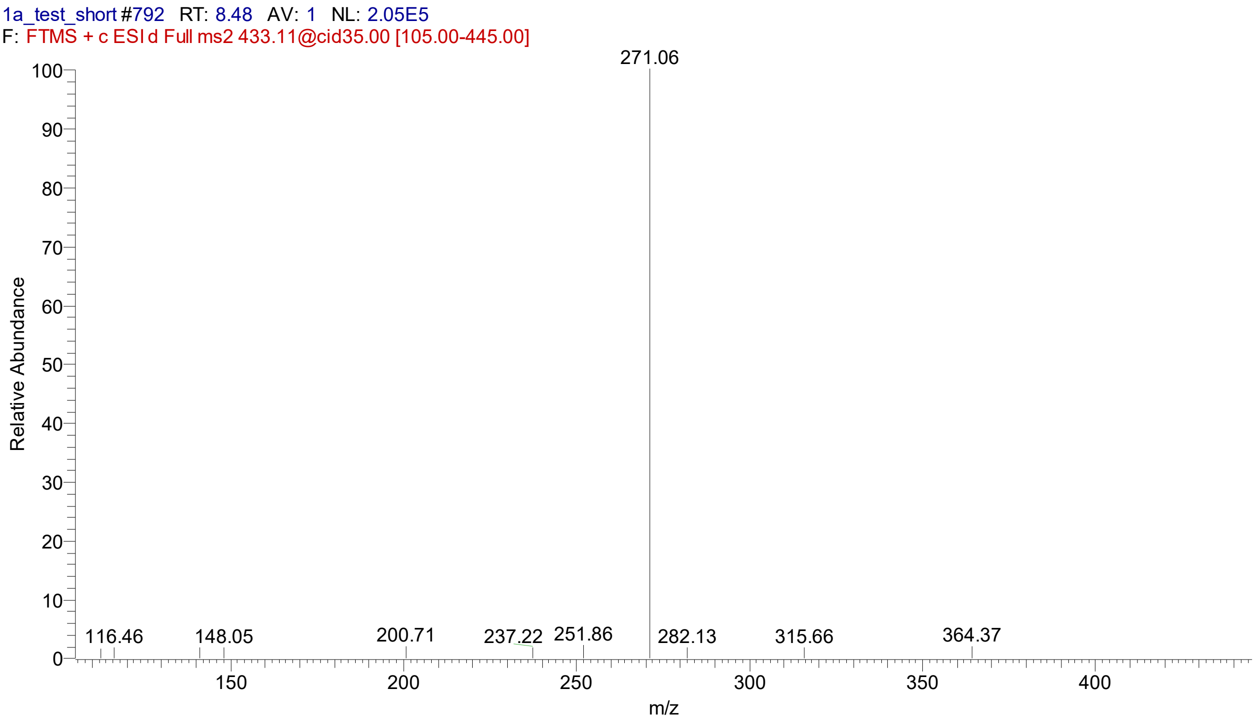

Figure S2. MS/MS spectra of compound **4** (pelargonidin 3-*O*-glucoside)

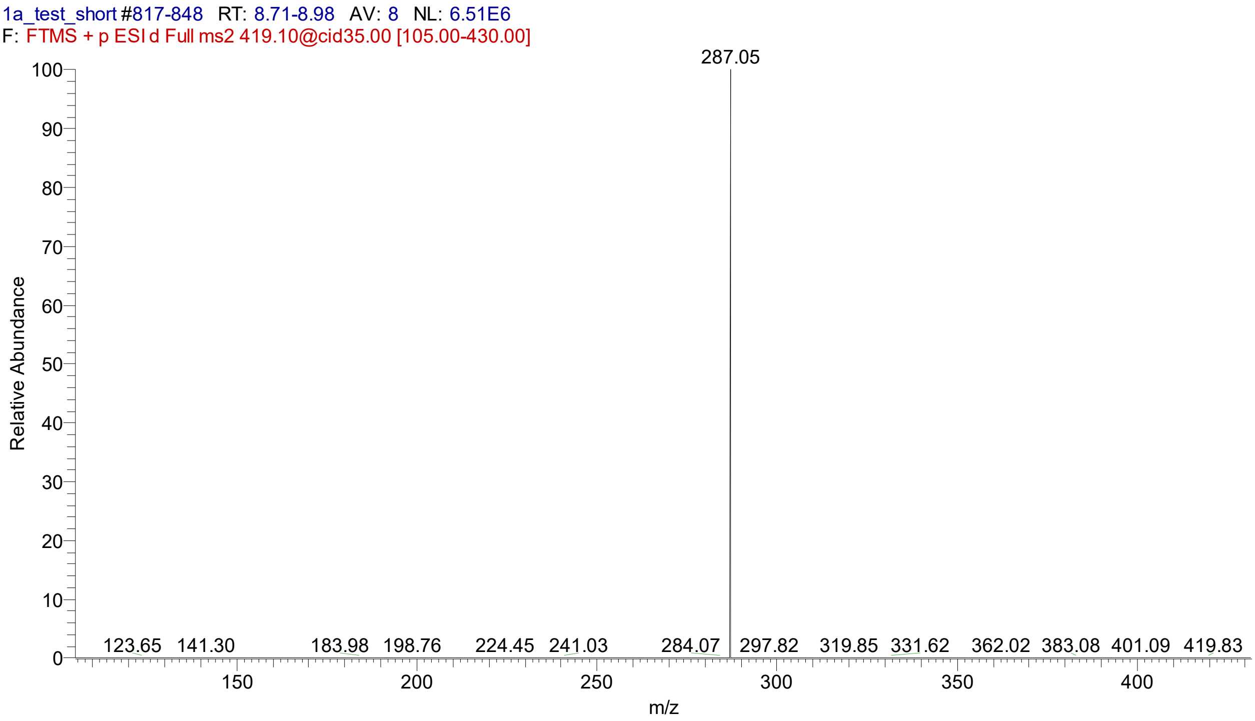

Figure S3. MS/MS spectra of compound **6** (cyanidin xyloside)

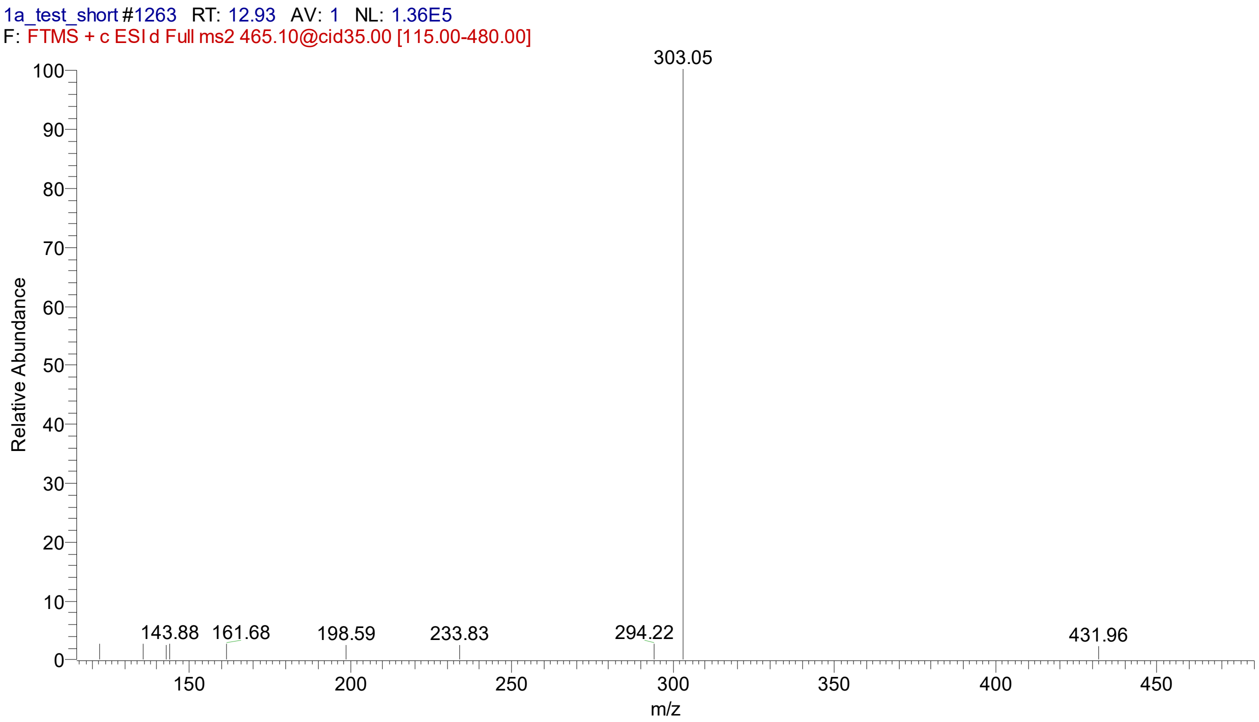

Figure S4. MS/MS spectra of compound **13** (delphinidin 3-*O*-glucoside)

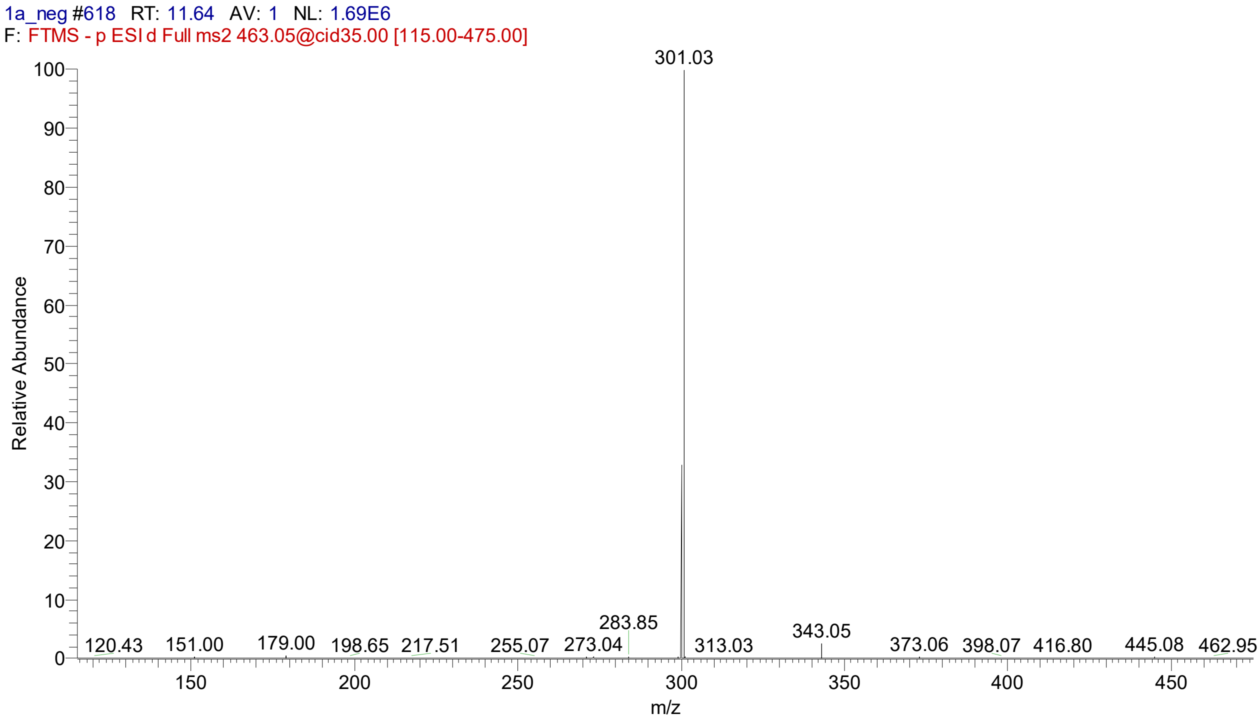

Figure S5. MS/MS spectra of compound **14** (quercetin 3-*O*-glucoside)

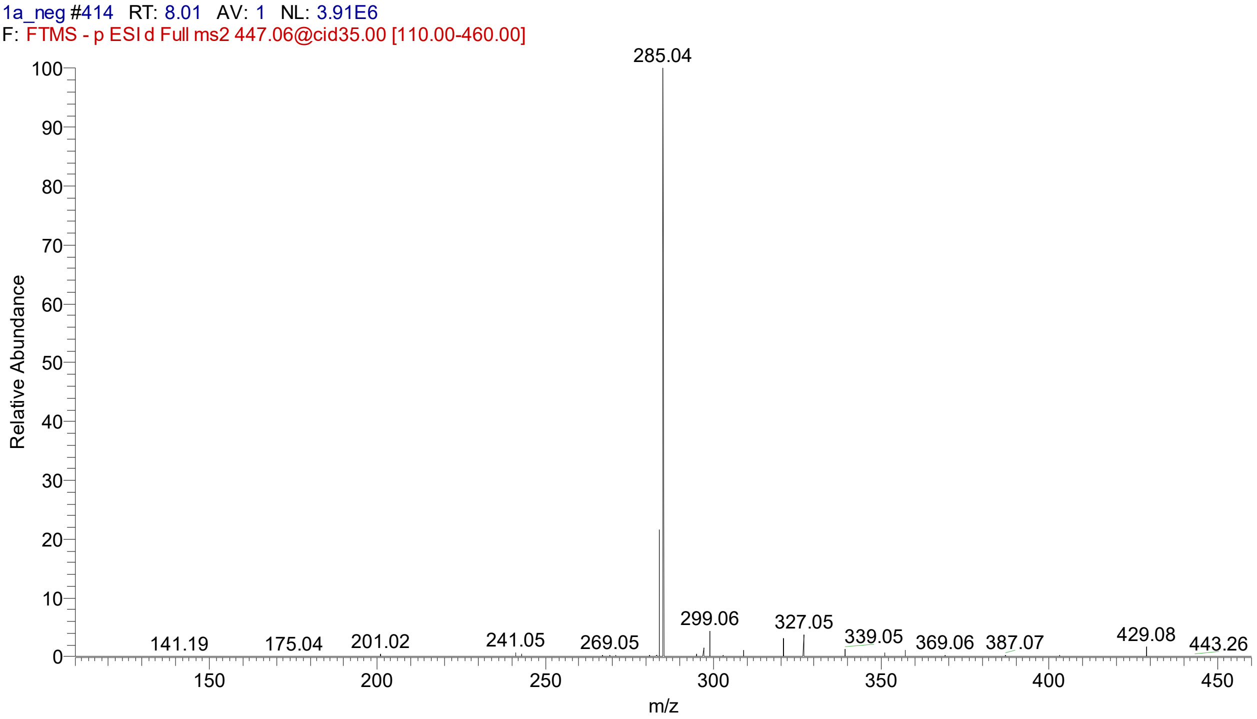

Figure S6. MS/MS spectra of compound **15** (kaempferol 3-*O*-glucoside)
